# The brain-derived neurotrophic factor (BDNF) val66met polymorphism differentially affects performance on subscales of the Wechsler Memory Scale – Third Edition (WMS-III)

**DOI:** 10.3389/fpsyg.2015.01212

**Published:** 2015-08-17

**Authors:** Yvette N. Lamb, Christopher S. Thompson, Nicole S. McKay, Karen E. Waldie, Ian J. Kirk

**Affiliations:** School of Psychology, Faculty of Science, The University of Auckland, AucklandNew Zealand

**Keywords:** neurogenetics, BDNF, memory, recall, recognition

## Abstract

Single nucleotide polymorphisms in the brain-derived neurotrophic factor (*BDNF*) gene and the catechol-*O*-methyltransferase (*COMT*) gene influence brain structure and function, as well as cognitive abilities. They are most influential in the hippocampus and prefrontal cortex (PFC), respectively. Recall and recognition are forms of memory proposed to have different neural substrates, with recall having a greater dependence on the PFC and hippocampus. This study aimed to determine whether the *BDNF* val^66^met or *COMT* val^158^met polymorphisms differentially affect recall and recognition, and whether these polymorphisms interact. A sample of 100 healthy adults was assessed on recall and familiarity-based recognition using the Faces and Family Pictures subscales of the Wechsler Memory Scale – Third Edition (WMS-III). *COMT* genotype did not affect performance on either task. The *BDNF* polymorphism (i.e., met carriers relative to val homozygotes) was associated with poorer recall ability, while not influencing recognition. Combining subscale scores in memory tests such as the WMS might obscure gene effects. Our results demonstrate the importance of distinguishing between recall and familiarity-based recognition in neurogenetics research.

## Introduction

Individual differences in the memory ability of healthy individuals are ubiquitous, readily observed both within the laboratory and without. A single nucleotide polymorphism (SNP) found in the gene coding for brain-derived neurotrophic factor (BDNF) has been implicated in variation in mnemonic ability (e.g., Egan et al., 2003; Hariri et al., 2003). Similarly, individual differences in a range of cognitive skills have been attributed partly to a SNP in the gene coding for catechol-*O*-methyltransferase (COMT; e.g., Egan et al., 2001; de Frias et al., 2004). The present study investigated the effects of the *BDNF* val^66^met and *COMT* val^158^met polymorphisms on recall and recognition, two neurally dissociable forms of memory.

When researching the potential genetic correlates of memory performance, it is necessary to distinguish between forms of memory. Previous research in this area has often used memory scores that collapse across different forms of memory. However, it is probable that forms of memory are influenced by unique clusters of genes (Nilsson, 2000). One major qualitative division of memory exists between recall and familiarity judgments (Mandler, 1980, 2008). Familiarity-based memory, a component of recognition, is the capacity to judge the extent to which a stimulus has previously occurred. This ability does not necessitate the retrieval of specific information concerning the context in which the previous encounters took place (Perfect et al., 1996). Recollection, on the other hand, entails the retrieval of precise identifying characteristics of the stimulus, or contextual information such as concepts with which the stimulus was previously associated.

Aggleton and Brown (1999) proposed that these memory functions are the products of two limbic loops that are, to a large extent, functionally independent. The recall of episodic or episodic-like information is achieved by a network of structures involving the hippocampus and the anterior thalamic complex. Familiarity judgments were proposed to be accomplished by a second network which instead incorporates the perirhinal cortex and the mediodorsal thalamus. Animal studies (e.g., Morris et al., 1982; Meunier et al., 1993; Steele and Rawlins, 1993; Duva et al., 1997; Bussey et al., 1999) and neuroimaging studies of humans (e.g., Squire et al., 1992; Schacter et al., 1996; Kirk et al., 2004; Ryan et al., 2008; Kafkas and Montaldi, 2012) have provided empirical support for Aggleton and Brown’s hypotheses, although there is some recent evidence that the mediodorsal thalamus plays a role in recall (e.g., Pergola et al., 2012, 2013).

While most aspects of Aggleton and Brown’s (1999) model have received considerable support, one criticism has been that Aggleton and Brown (1999) do not fully acknowledge the particular importance of the prefrontal cortex (PFC) in recall (Knowlton, 1999; Parker, 1999). A review by Davidson et al. (2006) suggests that the PFC may be more crucial for recall than recognition, although further research into this is necessary. The greater importance of the PFC in recall is consistent with the role this region is thought to play in binding diverse pieces of information together (Fernández and Tendolkar, 2001). From research implicating frontal and medial temporal formations in memory processes, it follows that genes affecting the structure and function of these regions could also influence memory performance.

Part of the neurotrophin family, BDNF is a small, dimeric signaling protein (Lessmann et al., 2003). BDNF promotes neuronal growth and differentiation whilst the peripheral and central nervous systems develop (Poo, 2001; Huang and Reichardt, 2003). Although the functions of BDNF in the adult brain are less understood (Cunha et al., 2010), BDNF has been found to encourage neurogenesis in the mature dentate gyrus (Sairanen et al., 2005; Scharfman et al., 2005; Thakker-Varia et al., 2014) and striatum (Mohapel et al., 2005). BDNF also appears to play a vital role in long-term potentiation (LTP; Poo, 2001; Panja and Bramham, 2014), the long-lasting enhancement of synaptic efficacy that is thought to underlie memory and learning (Bliss and Collingridge, 1993; Cooke and Bliss, 2006). Egan et al. (2003) proposed that it is through the role of BDNF in LTP that BDNF secretion impacts memory and learning (see Lamb et al., 2014 for a recent review).

The *BDNF* val^66^met polymorphism produces a non-conservative substitution of a valine with a methionine at codon 66 of this gene (Egan et al., 2003; Chen et al., 2004). In a population of European ancestry, 64% of individuals are val homozygotes (val/val), another 3% are met homozygotes (met/met), and the 34% that remain are heterozygotes (val/met; HapMap-CEU). The presence of the met allele has been associated with decreased activity-dependent secretion of BDNF and abnormal intracellular trafficking of the protein (Egan et al., 2003). In accordance with BDNF expression being maximal in the hippocampus (Murer et al., 2001), three meta-analyses have reported that the met allele is associated with lower hippocampal volume (Hajek et al., 2012; Kambeitz et al., 2012; Molendijk et al., 2012). It should, however, be noted that a later meta-analysis by Harrisberger et al. (2014) found no evidence of *BDNF* genotype affecting hippocampal volume, suggesting previous effects may have been overestimated.

Hariri et al. (2003) demonstrated that individuals carrying a met allele show less hippocampal activation during memory encoding and retrieval than val homozygotes, which may reflect impaired synaptic events in the met carriers. A weaker memory trace may be formed, thus accounting for their poorer subsequent performance on the hippocampal-dependent memory task. While the cumulative literature does suggest that the met allele is detrimental to memory (see Kambeitz et al., 2012 for a meta-analysis), a number of empirical studies have not detected an association between genotype and memory performance (e.g., Hansell et al., 2007; van Wingen et al., 2010). This inconsistency may in part reflect differences in the forms of memory assessed and the extent to which they are dependent on the hippocampus.

As with the neurotrophins, neurotransmitters and the proteins that regulate them are integral to neurodevelopment and cognition. The metabolism of released dopamine is catalyzed by COMT, with the degradation decreasing levels of this neurotransmitter within the synapse (Egan et al., 2001). Insufficient dopamine has been implicated in deficits across a range of cognitive domains (Nieoullon, 2002), including memory (e.g., Brozoski et al., 1979; Cai and Arnsten, 1997). Interestingly, excessive dopaminergic activity appears to also be detrimental for the memory functions of the PFC (e.g., Cai and Arnsten, 1997; Zahrt et al., 1997). Xu et al. (2009) demonstrated that when dopamine is significantly increased in mice, the induction of LTP in the PFC is eroded rather than facilitated.

The activity and thermal stability of the COMT enzyme are influenced by a common SNP located on the coding region of the *COMT* gene (Lotta et al., 1995; Lachman et al., 1996). This val^158^met polymorphism involves a valine being switched for a methionine. In a European population, 29% are homozygous for the val allele (val/val), 25% are homozygous for the met allele (met/met) and the 46% are heterozygotes (val/met; HapMap-CEU). At body temperature, the met allele is associated with almost four times more COMT activity than the val allele (Lachman et al., 1996). Met homozygotes would thus be expected to have slower dopamine inactivation than val homozygotes. The alleles appear to be codominant, with heterozygotes displaying an intermediate phenotype (Chen et al., 2004).

*COMT* genotype has been found to affect gray matter levels in the hippocampus and dorsolateral PFC (Honea et al., 2009). Functional differences have also been observed. In an fMRI study by Egan et al. (2001), PFC blood oxygenation level dependent (BOLD) response during a working memory task differed as a function of genotype. The greatest BOLD response was seen in the val homozygotes and may indicate a less efficient system. This differential PFC response has been replicated in a number of later studies (e.g., Mattay et al., 2003; Meyer-Lindenberg et al., 2006; Jasper et al., 2015). As there are few dopamine transporters in the PFC, variation in COMT function could be particularly influential on activity in this region (McIntosh et al., 2007).

*COMT* genotype has been demonstrated to predict variation in executive function (e.g., Egan et al., 2001; Malhotra et al., 2002; Mitaki et al., 2013) and processing speed (e.g., Bilder et al., 2002), with the met allele typically being associated with superior performance (although a meta-analysis by Barnett et al., 2008 suggests genotypic effects may be smaller than initially thought). A number of studies have reported met homozygotes to have an advantage on memory tasks when compared to val carriers (e.g., de Frias et al., 2004; Enoch et al., 2009; Raz et al., 2009). de Frias et al. (2004) found that when episodic memory was broken down into its elements of recall and recognition, a significant difference between genotypes was only present for the recall component. This differential effect demonstrates that the polymorphism could have some degree of memory specificity, possibly driven by the relative involvement of the PFC.

The present study aimed to examine both recall and recognition performance in the same group of participants, to determine (1) whether the *BDNF* val^66^met polymorphism differentially influences performance on recall and recognition tasks; (2) whether the *COMT* val^158^met polymorphism differentially influences performance on recall and recognition tasks; and (3) whether the *BDNF* val^66^met and *COMT* val^158^met polymorphism interact to influence either recall or recognition performance. Based on the particular influence of the *BDNF* val^66^met polymorphism on the hippocampus and the specific dependency of recall on the hippocampus, it was hypothesized that the *BDNF* polymorphism would influence recall but not familiarity-based recognition. On the recall task, val homozygotes were expected to outperform those with the met allele, thus replicating the results of past research on hippocampal-dependent memory (e.g., Egan et al., 2003; Hariri et al., 2003). As the influence of the *COMT* val^158^met polymorphism appears to be highest in the PFC, *COMT* genotype was also predicted to solely affect performance on the recall task. On this task, met homozygotes were expected to perform better than individuals with the val allele, replicating de Frias et al. (2004).

Brain-derived neurotrophic factor plays a pivotal role in the development of dopaminergic-related systems (Zhou et al., 1994), while COMT levels affect the structure of frontal and limbic regions (e.g., Honea et al., 2009). Furthermore, BDNF (Poo, 2001) and COMT (Jacobsen et al., 2010) both influence forms of LTP and a study by Witte et al. (2012) has noted that the *BDNF* and *COMT* polymorphisms interact to impact on cortical plasticity. A *BDNF* and *COMT* interaction has also been recently reported for immediate recall in older adults (Stuart et al., 2014). Consequently, it was hypothesized that an interaction between the *BDNF* val^66^met and *COMT* val^158^met polymorphisms might be found for recall performance.

## Materials and Methods

### Participants

A sample of 100 healthy university students aged between 18 and 42 years (*M* = 23.3, SD = 4.0) participated in this study. Of these participants, 64 were female. Participants had either normal or corrected-to-normal vision. All participants gave their informed consent for inclusion in this study and the University of Auckland Human Subjects Ethics Committee approved all study procedures.

### Genotyping

#### DNA Collection

Participants were asked to give a small blood sample or saliva sample. Blood sample collection was performed with sterile procedures. Saliva samples were collected using Oragene-DNA Self Collection kits in a manner consistent with the manufacturer’s instructions.

#### DNA Extraction

DNA was extracted from the blood samples following the method outlined by Miller et al. (1988) and from the saliva samples following the method given by Nishita et al. (2009). All resultant DNA samples were resuspended in Tris-EDTA buffer and were quantified used Nanodrop ND-1000 1-position spectrophotometer (Thermo Scientific).

#### DNA Amplification

The DNA samples were all diluted to 50 ng/μL. A modified version of the method described by Erickson et al. (2008) was used for the DNA amplification. Amplification was carried out on the 113 bp polymorphic *BDNF* fragment, using the primers BDNF-F 5-GAG GCT TGC CAT CAT TGG CT-3 and BDNF-R 5-CGT GTA CAA GTC TGC GTC CT-3. Amplification of the 176 bp polymorphic *COMT* fragment used the primers COMT-F 5-TCA CCA TCG AGA TCA ACC CC-3 and COMT-R 5-GAA CGT GGT GTG AAC ACC TG-3. Polymerase chain reaction (PCR) was conducted using 10X Taq buffer (2.5L μL), Taq polymerase (0.125 μL), dNTPs (5 nmol), primers (10 pmol each), Q solution (5 μL), and DNA (100 ng) made up to 25 μL with dH_2_O. The PCR conditions consisted of denaturation at 95°C for 15 min, 30 cycles on a ThermoCycler (involving denaturation at 94°C for 30 s, annealing at 60°C for 30 s, and extension at 72°C for 30 s) and a final extension at 72°C.

#### Enzyme Digestion

For *BDNF*, PCR product (6.5 μL) was incubated with Pm1l at 37°C overnight. For *COMT*, PCR product (8 μL) was incubated with N1aIII at 37°C for 1 h. The digestion products were analyzed using a high-resolution agarose gel (4%) with a Quick Load 100 bp ladder (BioLabs) and a GelPilot Loading Dye (QIAGEN). After immersion in an ethidium bromide solution for 10 min, DNA was visualized under ultraviolet light.

#### Genotyping

For *BDNF*, enzyme digestion resulted in a 113 bp fragment for the met^66^ allele, which was cut into 78 and 35 bp fragments for the val^66^ allele. For *COMT*, digestion resulted in bands of 82, 54 and 41 bp for the val^158^ allele and the 82 bp fragment was cut into 64 and 18 bp fragments for the met^158^ allele. This was as described by Erickson et al. (2008).

### Memory Measurements

Familiarity and recall performance were assessed using two subtests from the Wechsler Memory Scale – Third Edition (WMS-III; Wechsler, 1997b). These subtests were the Faces and Family pictures tasks, each of which tap into visual memory. In the Faces subtest, participants were presented with 24 images of faces that they were requested to remember. The faces were presented serially for 2 s each. Immediately after being presented with this list, participants were shown 48 faces, half of which they had just seen and the rest of which were novel. For each of these, participants were required to make a judgment as to whether or not they had previously been shown it. Raw Faces scores were converted into percentage correct for each participant.

In the Family Pictures task, participants were first introduced to images of a fictional family consisting of seven members. They were then presented with four scenes in turn, shown for 10 s each. Within each of these scenes, up to four members of the fictional family appear engaged in various activities in unique spatial locations. Immediately subsequent to the viewing of these, participants were asked set questions that assessed their memory of the scenes. These questions related to the activities and locations of each character. Unlike the Faces subtest, the Family Pictures subtest necessitates recall, with contextualized details of the scenes being retrieved from memory. Raw Family Pictures scores were converted into percentage correct for each participant.

### Data Analysis

#### Data Preparation

Observed *BDNF* genotypes did not differ significantly from those predicted by Hardy Weinberg equilibrium (*χ*^2^ = 0.849, *p* > 0.05). Of the 100 participants, 53 (53.0%) were val (G) homozygotes, 10 (10.0%) were met (A) homozygotes and 37 (37.0%) were heterozygotes (val/met; G/A). *BDNF* genotypes were dichotomised into val homozygotes and met allele carriers for analysis. While research would ideally distinguish between the *BDNF* val/met and met/met genotypes, this is often not practical due to the rarity of met homozygotes and low sample sizes. Consequently, numerous previous studies have combined heterozygotes and met homozygotes in this manner (e.g., Pezawas et al., 2004; Erickson et al., 2008), still detecting significant differences.

For similar reasons, *COMT* genotypes were dichotomised into met homozygotes and val allele carriers. A number of prior cognitive studies have found only the *COMT* met homozygotes to significantly differ from the other genotypes, supporting the decision of grouping the heterozygotes and val homozygotes together (e.g., Malhotra et al., 2002; Tsai et al., 2003). *COMT* genotypes in the present study did not differ significantly from those predicted by Hardy Weinberg equilibrium (χ^2^ = 0.998, *p* > 0.05). Of the 100 participants, 27 (27.0%) were val (G) homozygotes, 28 (28.0%) were met (A) homozygotes and 45 (45.0%) were heterozygotes (val/met; G/A).

#### Statistical Analyses

A MANOVA was conducted on the Family Pictures (recall) scores and Faces (recognition) scores, with *BDNF* genotype (val/val and met allele) and *COMT* genotype (val allele and met/met) as the between-subjects independent variables.

Mean recall and recognition scores for *BDNF* and *COMT* genotypes are shown in **Table 1**.

**Table 1:** Mean recall and recognition scores for brain-derived neurotrophic factor (BDNF) and catechol-*O*-methyltransferase (COMT) genotypes.

Form of memory	*BDNF*	*COMT*	Mean	SE	*N*
Recall	Val/Val	Val allele	76.8	2.08	40
		Met/Met	81.8	3.65	13
		Total	79.3	2.10	53
	Met allele	Val allele	75.3	2.32	32
		Met/Met	68.8	3.40	15
		Total	72.0	2.06	47
	Total	Val allele	76.1	1.56	72
		Met/Met	75.3	2.49	28
		Total	75.8	1.34	100
Recognition	Val/Val	Val allele	79.1	1.71	40
		Met/Met	81.1	3.01	13
		Total	80.1	1.73	53
	Met allele	Val allele	78.8	1.92	32
		Met/Met	82.6	2.80	15
		Total	80.7	1.70	47
	Total	Val allele	78.9	1.29	72
		Met/Met	81.9	2.05	28
		Total	79.8	1.08	100

## Results

**FIGURE 1:**
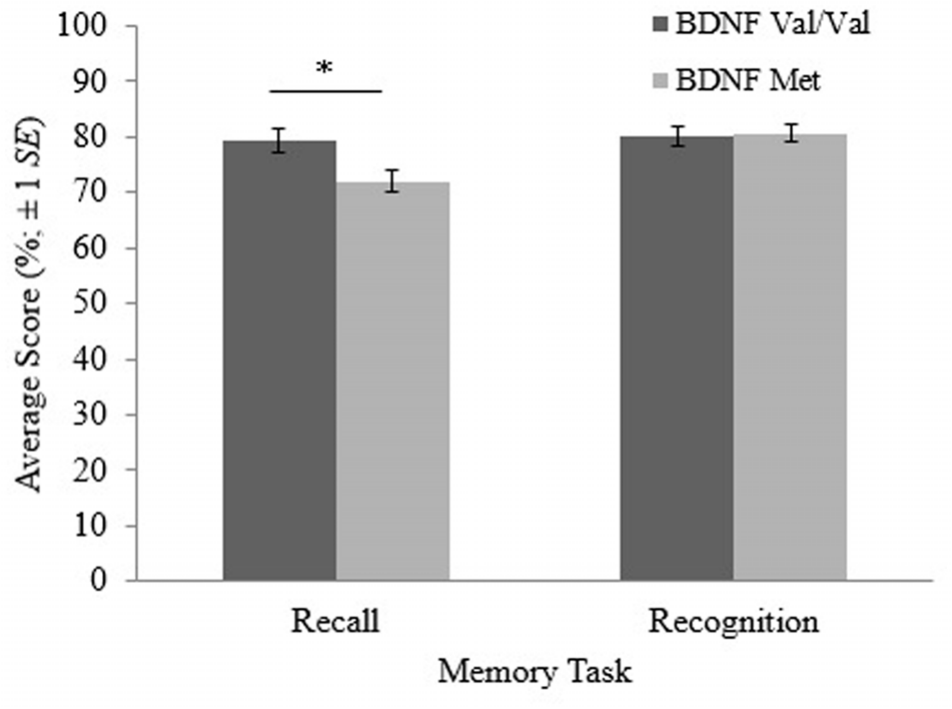
**Recall and recognition scores for participants with the brain-derived neurotrophic factor (*BDNF*) val/val genotype and participants with at least one copy of the *BDNF* met allele.** The error bars are based on ±1 SE. *p < 0.05.

Results of the MANOVA are shown in **Table 2**. The MANOVA revealed a significant main effect of *BDNF* genotype on recall performance [*F*_(1,96)_ = 6.204, *p* = 0.014]. This main effect is shown in **Figure 1**. On average, *BDNF* val homozygotes (*M* = 79.3, SE = 2.10) attained significantly higher recall scores than met allele carriers (*M* = 72.0, SE = 2.06).

**Table 2:** MANOVA for recall (Family Pictures) and recognition (Faces) scores with BDNF genotype and COMT genotype as the between-subjects variables.

Source		*F*	df	*P*
*BDNF* genotype	Recall	6.204*	1	0.014
	Recognition	0.068	1	0.795
*COMT* genotype	Recall	0.068	1	0.795
	Recognition	1.477	1	0.227
BDNF*COMT	Recall	3.864	1	0.052
	Recognition	0.143	1	0.706
Error			96	

*p < 0.05.

A two-way interaction between *BDNF* and *COMT* genotype on recall scores was approaching significance [*F*_(1,96)_ = 3.864, *p* = 0.052]. It should be noted that neither this interaction trend nor the main effect of *BDNF* on recall appear to have been driven by influential outliers. Data screening results were not consistent with the presence of influential outliers and Shapiro–Wilk tests indicate recall scores were sufficiently normally distributed for each *BDNF* and *COMT* genotype combination (*p* > 0.05).

No other effects or interactions were significant for recall or recognition.

## Discussion

Support was found for the hypothesis that the *BDNF* val^66^met polymorphism would differentially affect recall and familiarity-based recognition. As predicted, there was a main effect of *BDNF* genotype for recall scores, with val homozygotes significantly outperforming those with a copy of the met allele. In contrast, there was no effect of *BDNF* genotype on recognition scores. The superior performance of val homozygotes on the recall task replicates previous studies that have assessed hippocampal-dependent memory (e.g., Egan et al., 2003; Hariri et al., 2003). Similarly, the lack of effect on familiarity-based recognition reproduces the null effects reported by van Wingen et al. (2010). The differential effect of the *BDNF* polymorphism on recall and recognition suggests that *BDNF* is less influential on extra-hippocampal structures and processes than it is on those of the hippocampus. This interpretation is consistent with research showing the anatomical and physiological effects of BDNF to be particularly salient in the hippocampus (e.g., Hofer et al., 1990), as well as Hariri et al. (2003) demonstration of *BDNF* genotype having a hippocampal-specific impact on activation levels. Consequently, the results from the present study may help explain some of the inconsistencies in the existing literature (see Mandelman and Grigorenko, 2012, and Kambeitz et al., 2012 for recent meta-analyses), in that *BDNF* is unlikely to be implicated in memory in studies where memory is assessed solely on familiarity judgments.

The differential effect on recall and recognition found in the present study has implications for theories of memory, particularly that of Aggleton and Brown (1999). Aggleton and Brown’s distinction between recall and familiarity-based recognition has been criticized on the grounds that the importance of recollection in familiarity-based recognition is widely acknowledged, with the overlap between recall and familiarity-based recognition rendering a neural dissociation between these forms of memory invalid (e.g., Mayes et al., 1999). The results of the present study suggest that the difference between recall and recognition is sufficient to have practical consequences for research, as well as possible clinical applications. A distinction between recall and familiarity-based recognition should be considered by researchers investigating the genetics and neural processes involved in memory.

The hypothesis that the *COMT* val^158^met genotype would differentially affect recall and recognition performance was not supported in the present study. *COMT* genotype affected neither recall nor recognition. This is inconsistent with previous studies that have found *COMT* genotype to have consequences for memory. Research by de Frias et al. (2004) reported that the *COMT* met allele was beneficial for recall performance, while not affecting recognition. The failure of the present study to replicate this result may be a consequence of differences between the participant samples. de Frias et al. (2004) research sample consisted of older participants, whereas the present study examined the performance of young adults. Furthermore, de Frias et al. (2004) study only involved male participants. Research indicates that the impacts of the *COMT* polymorphism on cognition can vary with age (e.g., Nagel et al., 2008), and that sex differences might be present (e.g., O’Hara et al., 2006). It should also be noted that meta-analyses (e.g., Barnett et al., 2008) suggest the effects of *COMT* on memory and other forms of cognition may not be as large as initially thought. Furthermore, other genetic variants affecting the dopaminergic system, such as the dopamine receptor D1 (*DRD1*) and D2 (*DRD2*) SNPs, should ideally be included in studies looking at the effects of *COMT*. When studied in isolation the effects of a single polymorphism on memory may be obscured (Gosso et al., 2008).

While we found a trend toward *BDNF* and *COMT* genotypes interacting to affect recall, this did not reach significance. It is possible that our study lacked the statistical power necessary to detect an interaction effect, due to having insufficient participants with certain *BDNF* and *COMT* genotypic combinations. While there is pre-existing evidence that *BDNF* genotypes influence levels of LTP induction (Thompson et al., 2013) and may interact with *COMT* genotypes in doing so (Witte et al., 2012), an effect on this brain-based phenotype may not necessarily result in a robust cognitive phenotype. While Stuart et al. (2014) did detect a significant interaction between *BDNF* and *COMT* on immediate auditory recall in older adults, their sample was larger and the effect size modest. Neurophysiological measures may be a more immediate reflection of the neurobiological effects of genes than more distal behavioral measures, as performance on behavioral tasks can be swayed by a multitude of additional factors including motivation, strategy use and attitude to assessment (Goldberg and Weinberger, 2004). Consistent with this, Kambeitz et al. (2012) found that the *BDNF* polymorphism has a weaker effect on memory performance than it does on hippocampal physiology.

Further studies replicating aspects of the present study would be constructive. The present study had several limitations, some of which were consequences of its small sample size. Due to having a limited number of participants of each genotype for each polymorphism, some genotypes were combined. As a result, this study was not capable of investigating the dosage effects that previous studies have reported to be evident across the three genotypes that result from each polymorphism (e.g., Egan et al., 2001; Beste et al., 2010; Wang et al., 2014). A study with a larger sample size would allow research into additional variables that could potentially influence the effects of these polymorphisms on memory. These variables include age, gender and general intelligence, as well as further genes (e.g., *DRD1* and *DRD2*).

There are also limitations associated with the use of the Faces and Family Pictures tasks from the WMS-III, tasks which have not been included in a more recent edition of the scale (WMS-IV; Wechsler, 2009) due in part to issues associated with their scoring systems (Pearson Clinical Assessment, 2009). In the present study, the Faces and Family Pictures tasks were scored according to the WMS-III Administration and Scoring Manual (Wechsler, 1997a). Many of our participants lost marks on the Family Pictures task due to misidentifying characters with similar appearances. As a consequence of these errors, they could not receive marks for any correct recall of the location and activity associated with that misidentified character. Therefore visual discrimination and recognition abilities also played a role in determining the scores participants received in the Family Pictures task, rather than it being a pure test of recalled associations. This is less than ideal and future research may look to replicate the present result with a more valid recall measure.

The present study contributes to our understanding of the genetic influences on normal memory variation in healthy young adults. It replicates and builds upon previous findings in demonstrating that the *BDNF* val allele benefits recall performance while not influencing familiarity-based recognition performance. The role of *BDNF* in the structure and function of the hippocampus in particular is consistent with the effect of the *BDNF* polymorphism being specific to hippocampal-dependent forms of memory such as recall. This differential effect on recall and recognition substantiates the legitimacy and desirability of distinguishing between these forms of memory when investigating the genetic underpinnings of memory. Combining subscale scores such as is usually done in the WMS will likely obscure the effects of the *BDNF* polymorphism. Sensitivity may be lost when collapsing across different cognitive phenotypes, contributing to inconsistencies in the literature.

## Conflict of Interest Statement

The authors declare that the research was conducted in the absence of any commercial or financial relationships that could be construed as a potential conflict of interest.
